# Prognosis of lung cancer with simple brain metastasis patients and establishment of survival prediction models: a study based on real events

**DOI:** 10.1186/s12890-022-01936-w

**Published:** 2022-04-27

**Authors:** Jiaying Yuan, Zhiyuan Cheng, Jian Feng, Chang Xu, Yi Wang, Zixiu Zou, Qiang Li, Shicheng Guo, Li Jin, Gengxi Jiang, Yan Shang, Junjie Wu

**Affiliations:** 1 Present Address: Department of Respiratory and Critical Care Medicine, Shanghai Changhai Hospital, the First Affiliated Hospital of Naval Medical University, Shanghai, 200433 China; 2 Department of Gastroenterology, Shanghai General Hospital, Shanghai Jiao Tong University School of Medicine, Shanghai, 200240 China; 3 Department of Thoracic Surgery, Shanghai Chest Hospital, Shanghai Jiaotong University, Shanghai, 200030 China; 4 Clinical College of Xiangnan University, Chenzhou, 423043 China; 5 School of Life Sciences, Fudan University, Shanghai, 200433 China; 6 Department of Respiratory and Critical Care Medicine, Shanghai East Hospital, Tongji University, Shanghai, 200120 China; 7 Department of Thoracic Surgery, Shanghai Changhai Hospital, the First Affiliated Hospital of Naval Military Medical University, Shanghai, 200433 China; 8 Department of General Medicine, the First Affiliated Hospital of Naval Medical University, Shanghai, 200433 China; 9 Department of Pulmonary and Critical Care Medicine, Fudan University, Shanghai, 200032 China; 10 Department of Pulmonary and Critical Care Medicine, Shanghai Geriatric Medical Center, Shanghai, 200032 China

**Keywords:** Lung cancer with simple brain metastasis, Prognostic analysis, SEER database, Nomogram

## Abstract

**Objectives:**

The aim of this study was to explore risk factors for the prognosis of lung cancer with simple brain metastasis (LCSBM) patients and to establish a prognostic predictive nomogram for LCSBM patients.

**Materials and methods:**

Three thousand eight hundred and six cases of LCSBM were extracted from the Surveillance, Epidemiology, and End Results (SEER) database from 2010 to 2015 using SEER Stat 8.3.5. Lung cancer patients only had brain metastasis with no other organ metastasis were defined as LCSBM patients. Prognostic factors of LCSBM were analyzed with log-rank method and Cox proportional hazards model. Independent risk and protective prognostic factors were used to construct nomogram with accelerated failure time model. C-index was used to evaluate the prediction effect of nomogram.

**Results and conclusion:**

The younger patients (18–65 years old) accounted for 54.41%, while patients aged over 65 accounted for 45.59%.The ratio of male: female was 1:1. Lung cancer in the main bronchus, upper lobe, middle lobe and lower lobe were accounted for 4.91%, 62.80%, 4.47% and 27.82% respectively; and adenocarcinoma accounted for 57.83% of all lung cancer types. The overall median survival time was 12.2 months. Survival rates for 1-, 3- and 5-years were 28.2%, 8.7% and 4.7% respectively. We found female (HR = 0.81, 95% CI 0.75–0.87), the married (HR = 0.80; 95% CI 0.75–0.86), the White (HR = 0.90, 95% CI 0.84–0.95) and primary site (HR = 0.45, 95% CI 0.39–0.52) were independent protective factors while higher age (HR = 1.51, 95% CI 1.40–1.62), advanced grade (HR = 1.19, 95% CI 1.12–1.25) and advanced T stage (HR = 1.09, 95% CI 1.05–1.13) were independent risk prognostic factors affecting the survival of LCSBM patients. We constructed the nomogram with above independent factors, and the C-index value was 0.634 (95% CI 0.622–0.646). We developed a nomogram with seven significant LCSBM independent prognostic factors to provide prognosis prediction.

## Introduction

Brain metastasis is one of the common metastatic mode of lung cancer [1]. It has the characteristics of advanced degree of malignancy, high mortality and difficulty in treatment. The main source of brain metastasis is also lung cancer [2]. And the brain is a specific metastatic organ of non-small cell lung cancer [3].

We studied the prognosis of lung cancer with simple brain metastasis (LCSBM), that was, lung cancer only had brain metastasis with no other organ metastasis. Some study showed the 5-year disease-free survival (DFS) and overall survival (OS) of non-small cell lung cancer were 82.4% and 85.4%, respectively [4]. It’s obvious that patients with LCSBM have a poorer prognosis [5]. However, the factors affecting prognosis have not been fully studied. At present, the most commonly used method is still the TNM staging system, with an unsatisfactory predictive effect [6]. Judging the prognosis is necessary in aspect of clinicians analyzing and evaluating the patients' conditions in order to better adjust the treatment strategies. Individualized prognostic diagnosis can lead to individualized treatment, which also has a good promotion effect on the development of precision medicine.

In view of this, an accurate and practical prognostic model is necessary. We selected data from the SEER database and established a nomogram to quantify the contribution of each risk factor to the prognosis. The SEER database is the abbreviation for the Surveillance, Epidemiology, and End Results (SEER) database. It is a program expanding over time to now include 18 registries, with information over enough to cover about 30% of the United States population, collecting data of their morbidity, mortality and survival [7]. A nomogram allows the model to be presented in an intuitive and simple form that can quickly achieve good results without any mathematical foundation or complex calculations.

The purpose of our study was to analyze the prognostic risk factors for LCSBM patients, and to establish a prediction model of patient prognosis in order to help clinical practice. This allows physicians to access the states of patients and lay the foundation for individualized treatment. What’s more, it will provide a credible explanation of the condition for patients and their families to avoid the lack of confidence and over-expectation, and facilitate the communication between the doctor and the patient.

## Material and methods

### Source

Data of patients with lung cancer brain metastasis from Surveillance, Epidemiology and End Results (SEER) database was searched and collected through queries using the latest version of the SEER 18 Registries Research Data (2010–2015), which was released in April 2017 with the SEER^*^Stat 8.3.5 software.

### Patient screening

Inclusion criteria:

1. Patients aged 18 years or older diagnosed with LCSBM (and younger than 80 at diagnosis).
2. Patients diagnosed between 2010 and 2015.
3. Patients diagnosed only primary neoplasms without multiple primary neoplasms elsewhere.
4. Patient diagnosed with pathological results.
5. Patients with complete follow-ups.
6. Patients died of LCSBM rather than other causes.

Exclusion criteria:

1. Unknown demographic information including diagnostic age, gender, marital status and race;
2. Unknown clinical information including primary site, TNM stage and grade;
3. Unknown treatment information including surgery and others;
4. Patients received chemotherapy or radiotherapy at diagnosis;
5. Patients with multiple primary tumors.

### Variable selection

There were 2 main types based on biology and treatment: small cell lung cancer and non-small cell lung cancer [8], and non-small cell lung cancer could be divided into squamous cell carcinoma, adenocarcinoma and large cell carcinoma. Since these sub-categories had an important impact on prognosis, our study discussed these types separately. Pathological grade was an important manifestation of the malignant degree of lung cancer. The advanced grade of pathology was closely related to the poor prognosis. According to the degree of differentiation of lung cancer, this study classified lung cancer into high differentiation (grade I), moderate differentiation (grade II), poorly differentiated (grade III) and undifferentiated (grade IV).The primary site was divided into: (1) Main bronchus; (2) Upper lobe, lung; (3) Middle lobe, lung and (4) Lower lobe, lung according to the anatomical structure. Location would be a meaningful taxonomy due to the different cellular structures. For the TNM staging system, the SEER database used the seventh edition of the TNM staging [9]. In order to maintain consistency in data measurement standards, this study also used the same staging criteria. Laterality was divided into left, right and others.

In terms of treatment, patients were divided into surgery group and non-surgical group. The SEER database did not contain information of radiotherapy and chemotherapy, so there was no relevant classification.

In terms of general conditions and epidemiological indicators, the study used X-tile software to obtain the best segmental age for diagnosis. And the race was divided into white, black and other (American Indian/AK Native, Asian/Pacific Islander). Marital status was classified as unmarried and married.

In addition, the patient's study endpoint was death or the deadline was March 2018.

### Statistical analyses

Univariate analysis and Cox proportional hazards model were performed by SPSS (v25.0). Prognostic overall survival was analyzed using Kaplan–Meier curves. Each of individual prognostic factors of LCSBM patients were analyzed by log-rank method. Introducing meaningful variables of single factor analysis into Cox proportional hazards model for multivariate analysis, the independent risk factors were obtained, *P* < 0.05 was statistically significant. By R Studio (v3.6.2), Independent risk factors were included in the accelerated failure time model to construct nomogram. C-index was used to access the predictive capacity of nomogram.

## Result

### Patient characteristics from SEER database

In our study, younger patients (18–65 years old) accounted for 54.41%, while patients aged over 65 accounted for 45.59%. Married people were slightly more than half. The major race was white, accounting for 78.24% of all selected patients, while blacks and others accounted for 13.40% and 8.36%, respectively. The ratio of male to female patients was about 1:1. The most common type of histology was adenocarcinoma, accounting for 57.83%. For laterality, left and right accounted for 41.93% and 58.07%, respectively.

Univariate analysis showed that the factors affecting the prognosis of LCSBM included the following factors (Table 1): age (*χ*^*2*^ = 163.16, *p* =  < 0.001), marital Status (*χ*^*2*^ = 43.985, *p* =  < 0.001), primary Site (*χ*^*2*^ = 10.727, *p* = 0.013), race (*χ*^*2*^ = 16.999, *p* =  < 0.001), surgery (*χ*^*2*^ = 184.795, *p* =  < 0.001), gender (*χ*^*2*^ = 31.99, *p* =  < 0.001), grade (*χ*^*2*^ = 71.301, *p* =  < 0.001), histologic type (*χ*^*2*^ = 98.416, *p* =  < 0.001), T Stage (*χ*^*2*^ = 58.295, *p* =  < 0.001) and N stage (*χ*^*2*^ = 25.029, *p* =  < 0.001) (Table 1).

**Table 1:** Clinicopathologic characteristics of patients

Clinicopathologic parameters	Number of cases		Average survival (month)	95% CI	*χ*^*2*^	*p*
	n	%				
*Age*					163.160	< 0.001
18–65	2071	54.41	15.061	14.107–16.014		
≥ 66	1735	45.59	8.806	8.070–9.542		
*Marital status*					43.985	< 0.001
Non-married	1786	46.93	10.377	9.550–11.205		
Married	2020	53.07	13.808	12.890–14.727		
*Primary site*					10.727	0.013
Main bronchus	187	4.91	9.0410	6.979–11.102		
Upper lobe, lung	2390	62.80	12.503	11.708–13.299		
Middle lobe, lung	170	4.47	13.994	10.790–17.198		
Lower lobe, lung	1059	27.82	11.686	10.520–12.852		
*Race*					16.999	< 0.001
White	2978	78.24	11.861	11.172–12.549		
Black	510	13.40	11.708	10.061–13.355		
Other	318	8.36	15.783	13.418–18.148		
*Surgery*					184.795	< 0.001
No surgery	3463	90.99	10.550	9.978–11.121		
Surgery	343	9.01	28.233	25.065–31.401		
*Gender*					31.990	< 0.001
Male	1970	51.76	10.804	10.008–11.600		
Female	1836	48.24	13.706	12.746–14.667		
*Grade*					71.301	< 0.001
I	140	3.68	19.020	14.870–23.170		
II	880	23.12	15.229	13.807–16.651		
III	2446	64.27	10.996	10.270–11.722		
IV	340	8.93	9.712	8.026–11.398		
*Laterality*					0.475	0.491
Left	1596	41.93	12.216	11.293–13.138		
Right	2210	58.07	12.153	11.318–12.988		
*Histologic type*					98.416	< 0.001
Squamous cell carcinoma	555	14.58	8.131	7.085–9.176		
Adenocarcinoma	2201	57.83	14.318	13.411–15.225		
Small cell lung cancer	335	8.80	9.832	8.156–11.507		
Large cell carcinoma	101	2.65	8.456	6.289–10.623		
Others	614	16.13	10.049	8.656–11.442		
*T stage*					58.295	< 0.001
T1	430	11.30	16.512	14.280–18.744		
T2	1283	33.71	13.424	12.311–14.537		
T3	1014	26.64	10.74	9.625–11.855		
T4	1079	28.35	10.234	9.219–11.248		
*N stage*					25.029	< 0.001
N0	1078	28.32	14.532	13.183–15.882		
N1	400	10.51	12.973	11.025–14.921		
N2	1778	46.72	11.123	10.302–11.943		
N3	550	14.45	10.118	8.739–11.496		
*Overall*	3806	100.00	12.234	11.604–12.864		

**Fig. 1:**
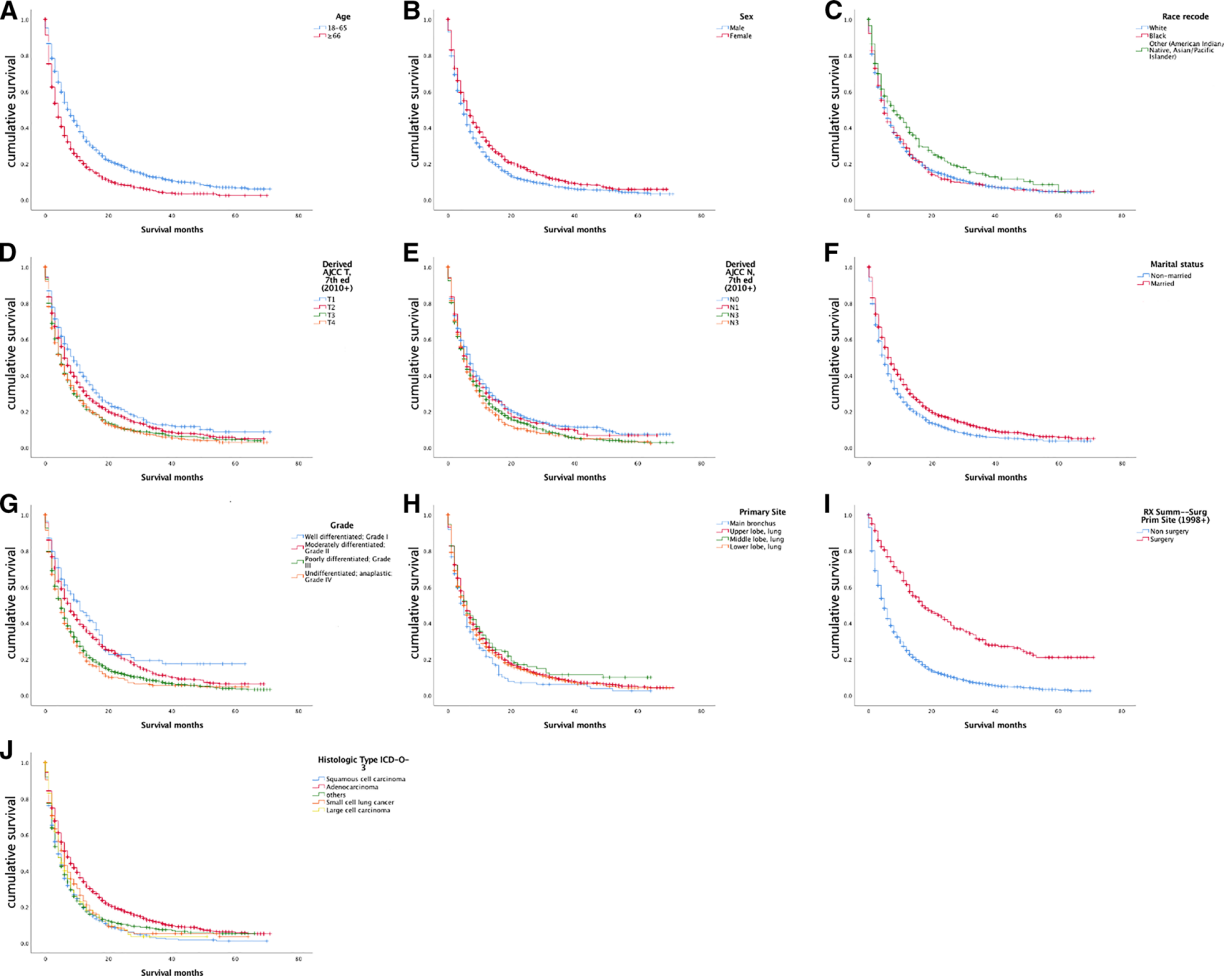
Charts from **A**–**J** are *Kaplan-Meier* Curve of prognostic factors. *Note*: figures refer to age, gender, race, T stage, N stage, marital status, grade, primary site, surgery, histologic type, respectively

The prognostic survival time of patients aged 18–65 (average survival time: 15.1 months, 95% CI 14.107–16.014) was better than that of patients aged 66 and over (average survival time: 8.8 months, 95% CI 8.070–9.542). The prognosis of unmarried patients (10.4 months) was worse than that of married (13.8 months) and the prognosis of males was worse than that of females (10.8 months vs 13.7 months). For the primary site, middle lobe of lung had the best prognosis, better than main bronchus; upper lobe and lower lobe (14.0 months vs 9.0 months vs 12.5 months vs 11.7 months) (Fig. 1).

### Multivariate analysis of prognostic factors

Introducing the significant factors of single factor analysis into Cox proportional risk model for multi-factor analysis and then we got independent prognostic factors as follows: age (HR = 1.506, 95% CI 1.402–1.617), marital status (HR = 0.804, 95% CI 0.749–0.864), gender (HR = 0.806, 95% CI 0.751–0.866), race (HR = 0.896, 95% CI 0.844–0.951), grade (HR = 1.185, 95% CI 1.123–1.251), T stage (HR = 1.092, 95% CI 1.054–1.132) and primary site (HR = 0.451, 95% CI 0.390–0.521) (Table 2).

**Table 2:** Independent factors for the prognosis of lung cancer brain metastasis

Independent risk factors	Regression coefficient	SE	*P*	HR	95% CI
Age	0.409	0.036	< 0.001	1.506	1.402–1.617
Marital status	− 0.218	0.036	< 0.001	0.804	0.749–0.864
Gender	− 0.215	0.037	< 0.001	0.806	0.751–0.866
Race	− 0.110	0.030	< 0.001	0.896	0.844–0.951
Grade	0.170	0.028	< 0.001	1.185	1.123–1.251
T stage	0.088	0.018	< 0.001	1.092	1.054–1.132
Primary site	− 0.797	0.074	< 0.001	0.451	0.390–0.521

As shown in Table 2, the protective factors included married, women, white people and primary site, while risk factors for poor prognosis include: higher age, advanced grade and advanced T stage.

### Development and verification of prediction model nomogram

**Fig. 2:**
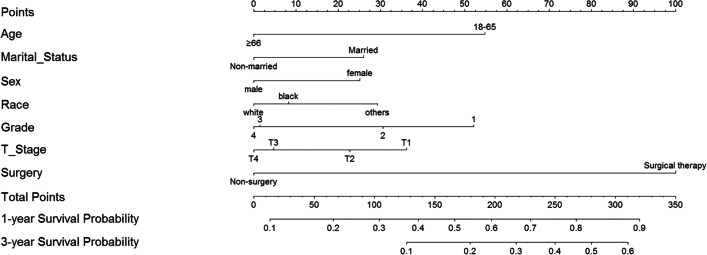
Nomogram for predicting 1- and 3-year *cancer-specific survival* of patients with lung cancer brain metastasis. Note: Grade: I = 1; II = 2; III = 3; IV = 4

**Fig. 3:**
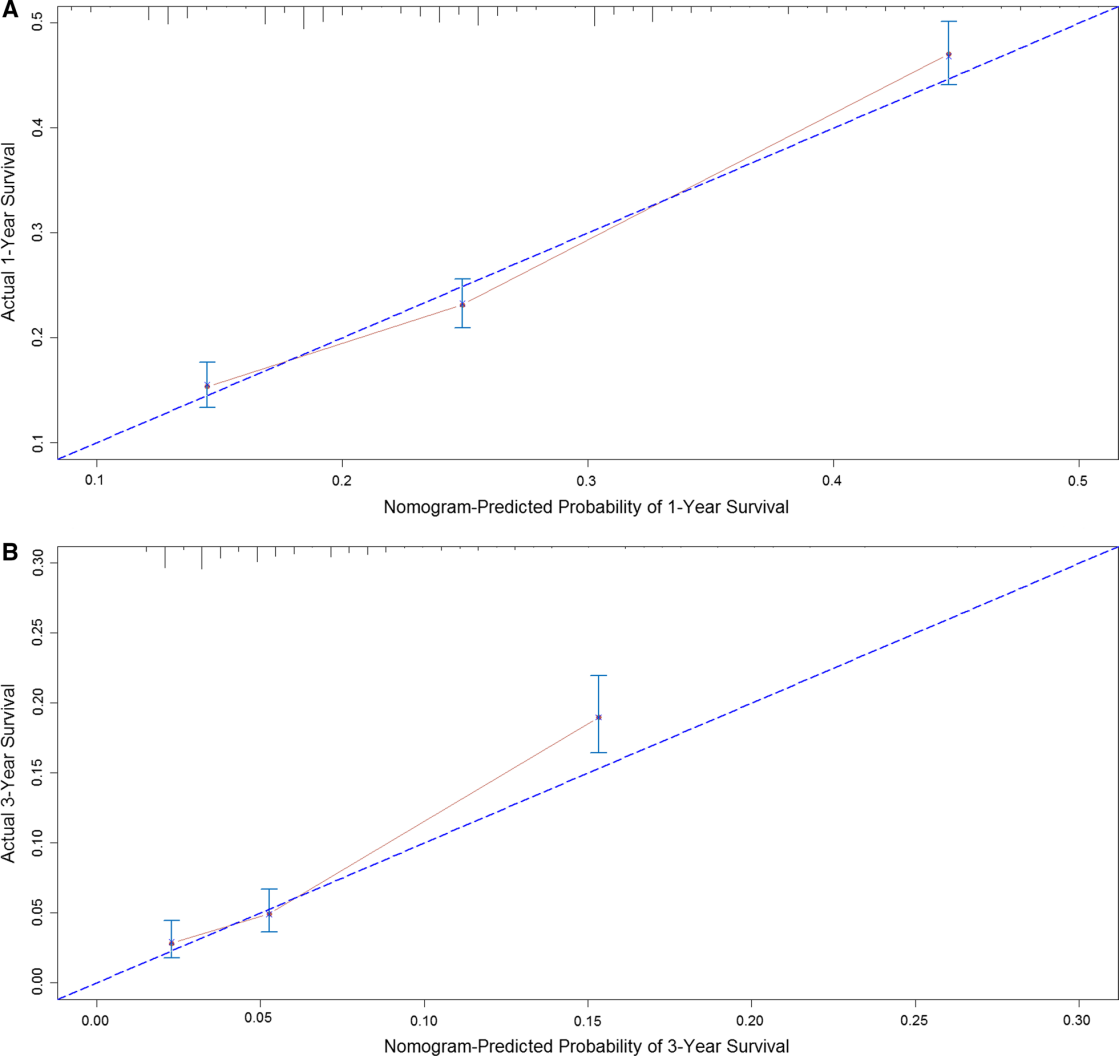
Nomogram model calibration curves. **A** 1-years calibration curves; **B** 3-years calibration curves. *Note*: The x-axis shows the nomogram predicted probability, and the y-axis gives the actual survival as estimated by the Kaplan–Meier method

With the results of multivariate analysis, we constructed a nomogram (Fig. 2). The risk factors introduced in the model were given different weights according to the degree of influence, and different scores were obtained according to the individual information of the patients. Adding the scores together to get the final score, and the prognostic prediction results could be found in the nomogram. Internally validation was done by discrimination and calibration method. The calibration plots showed correlation between observed OS and nomogram predicted OS. C-index of the predictive model in this study was 0.634 (95% CI 0.622–0.646), showing a good prediction effect (Fig. 3).

## Discussion

### Analysis of demographic results

We used the SEER database to obtain information of 3806 patients diagnosed with LCSBM, and extracted general status indicators, pathological indicators and treatment status indicators to comprehensively analyze the risk factors of LCSBM prognosis. This was the first nomogram predicting the prognosis of LCSBM patients, which could better optimize the diagnosis and treatment plan and help improve the prognosis of patients. At the same time, there were some deviations in this study, which would be discussed in the limitations of the study later.

In our study, the average survival time for patients with LCSBM was 12.2 months. Survival rates for 1-, 3- and 5-years were 28.2%, 8.7% and 4.7%, respectively. In general, lung cancer is more likely to occur in the old, but our study showed that the proportions of young people and that of old people diagnosed with LCSBM were similar, suggesting that young people might be more prone to brain metastasis, which deserved further study. In terms of race, white patients occupied a major part. Brain metastasis might be related to genes in combination with age and race.

The most common histological type was adenocarcinoma in this study, while main type of lung cancer was squamous cell carcinoma, indicating that brain tissue had more affinity for lung adenocarcinoma cells. It was showed that adenocarcinoma-associated genes were closely related to brain metastasis, and the specific small RNA associated with metastasis in lung adenocarcinoma had been identified [10]. Most patients with brain metastases were pathological grade III, because advanced malignant tumors were prone to brain metastasis, just like breast cancer [11].

For the primary site, the upper lobe tumors accounted for more than half, and lung cancer often occurred in the upper lobe, which was consistent.

### Risk factors for small intestinal neoplasms

Patients with LCSBM had some similarities with other types of lung cancer patients. First, the mean prognosis of the old group was worse than that of the young group, which was consistent with most studies [12, 13]. Second, Deng et al. reported that marital status was a prognostic factor for distant metastasis of non-small cell lung cancer [14]. Marriage as an important external environment might be psychologically reflected in the prognosis [15]. Third, the higher the histological grade, the stronger the malignancy and the worse the prognosis. In addition to our research, there was no literature reporting whether the prognosis of LCSBM was related to grade, but it had been reported that the prognosis of patients with breast cancer brain metastases was related to histologic grade [16]. Therefore, we believed that this conclusion was reasonable. Fourth, in aspect of histological classification, Miller did a research showing that prognosis of adenocarcinoma was better than that of squamous cell carcinoma in non-small cell lung cancer with brain metastasis [17]. Our study reconfirmed this result. As the T or N stage increased, the tumor progressed gradually, and the survival time gradually decreased, which was in line with clinical experience [6, 16, 18]. Our study found that from the initial stage to the end stage, the difference in prognosis between different TNM stages was only about half a year. It was obvious that LCSBM had high level of malignancy. Finally, surgical resection could effectively prolong the prognosis of patients [19, 20]. Although the data showed that surgery could prolong the prognosis of patients well, many patients with brain metastases did not meet the surgical indications so that patients who could be operated were few. As a result, our study included a large number of inoperable people to improve the adaptability of the model.

More importantly, our study suggested that LCSBM was a special type of lung cancer. First, race was an important factor influencing the prognosis of LCSBM. Thus, the prognosis of LCSBM patients might be related to genes. It had been shown that* EGFR* mutation was an independent predictor of probabilistic and prognostic factors for brain metastases (BM) and it was also an overall survival (OS)-positive predictor of BM patients [21]. Lee believed that the presence of* EGFR* activating mutations should be used as an indicator of prognosis in patients with lung adenocarcinoma and brain metastases [22]. And according to Fu P, race was an independent predictor of* EGFR* mutation [23]. This confirmed our point of view to some extent. Another study showed that *Robo1* was also a cancer-promoting gene that might promote the development and progression of lung cancer and lung cancer brain metastasis [24]. Nowadays, the novel immunotherapy about programmed death ligand-1 (PD-L1) and programmed death ligand-1 (PD-1) receptor is getting wide attention, and many clinical trials showed that the objective response rate in patients PD-L1–positive tumors obviously higher than in those with PD-L1–negative tumors, PD-L1 protein expression may become a novel biomarker in the future to guide the clinical use of immunotherapy [25, 26].* ALK* and* ROS1* rearrangements have been proved as oncogenic drivers. Tejas Patil et al., reviewed 579 patients with stage IV NSCLC, and the results showed that the incidence of brain metastases for treatment-naïve, stage IV* ROS1+* and* ALK+* NSCLC were both up to about 34% and with no difference across* ROS1, ALK, EGFR, KRAS, BRAF* or other mutations groups [27, 28].

In summary, genetic differences were likely to be the underlying cause of different prognosis; Second, gender was an independent factor affecting prognosis, and the average survival time of women is 26.9%, which was higher than that of males. This was consistent with the findings of non-small cell lung cancer, showing that male is the independent risk factors for shortening the prognosis [29]. Under long-term chemotherapy, female also had a better prognosis than male [30]. This suggested that genes and hormones were involved; Third, Li et al. collected tumor tissue samples from 118 patients with non-small cell lung cancer, and found that* EGFR* gene mutations and high copy number of genes were more common in female patients [31]. The above-mentioned* EGFR* gene was associated with brain metastasis of lung adenocarcinoma, so we believed that the gender difference in prognosis may be derived from the gene.

In general, lung cancer patients with simple brain metastases have a poor prognosis, but its prognostic risk factors are unclear. From the results, we have identified these: patients with simple brain metastasis may be related to genetic differences, and endocrine status may affect the prognosis of patients.

### Nomogram for small intestinal neoplasms

In some studies, TNM staging system did not yield a suitable prognosis [32]. TNM staging system is a general-purpose model which has distinct deviations for specific diseases, especially those with low morbidity. And nomogram is a good alternative [33], it can quickly and intuitively get the patients’ prognosis. Nowadays, nomogram is currently used in a variety of fields.

In our subject-related areas, current researches included prognostic analysis of tumor brain metastasis [34], survival analysis of non-small cell lung cancer (NSCLC) after surgical resection [33], and prognosis of NSCLC brain metastasis after surgery [35, 36], but there had not been a LCSBM prognostic prediction model. Therefore, it was necessary to examine prognostic risk factors for such patients and establish a reliable prediction model. Our model was proven to achieve reliable accuracy and to meet the needs of doctors and patients. Doctors could adjust the treatment according to the specific information of the patients and carry out targeted individualized treatment. At the same time, for patients, brain metastasis indicates a small amount of time. Accurate prognosis is responsible for patients, and it will become an important reference to treatment choice and psychological preparation. Therefore, in order to maximize the effectiveness of treatment and improve the prognosis and quality of life of patients, our model is meaningful.

### Insufficient study

Although the evidence in this test was sufficient, the argument was reasonable, and an innovative viewpoint was put forward, there were certain deficiencies. First of all, the data source of this trial is the SEER database. It collected the data of residents from different regions in the United States. To draw conclusions that can apply to another area, the data from the local should be used to validate the model in advance. Second, for the SEER database, it only included some common information such as age, race, gender, histology level, TNM staging, etc. However, there are many other risk factors influencing the prognosis, and it is impossible to exhaust the enumeration. Genomic status, protein expression, family history, etc. were all excluded, which would cause bias. Third, although the information on radiotherapy and chemotherapy has recorded in the database, it was not recommended for the construction of nomogram due to the incompleteness of the data and the bias was impacted by the patient's willing to treat as well. Fourth, the test was a retrospective analysis based on the database, with the limitations of the trial itself, requiring further validation of the prospective cohort study to obtain sufficient evidence.

## Data Availability

All data in this paper are from SEER database, and we guarantee that all clinical data are anonymous. Publicly available datasets were analyzed in this study. This data can be found here: https://seer.cancer.gov/.com.

## Abbreviations

LCSBM: Lung cancer with simple brain metastasis
DFS: Disease-free survival
OS: Overall survival
SEER: Surveillance, Epidemiology, and End Results
BM: Brain metastases
NSCLC: Non-small cell lung cancer
